# Which Behavior Change Techniques are Associated with Changes in Physical Activity, Diet and Body Mass Index in People with Recently Diagnosed Diabetes?

**DOI:** 10.1007/s12160-014-9624-9

**Published:** 2014-05-08

**Authors:** Nelli Hankonen, Stephen Sutton, A. Toby Prevost, Rebecca K. Simmons, Simon J. Griffin, Ann Louise Kinmonth, Wendy Hardeman

**Affiliations:** 1 Behavioural Science Group, Primary Care Unit, Department of Public Health and Primary Care, Institute of Public Health, University of Cambridge, Robinson Way, Cambridge, CB2 0SR UK; 2 Social Psychology Unit, Department of Social Research, University of Helsinki, PO Box 54, 00014 Helsinki, Finland; 3 The Primary Care Unit, Department of Public Health and Primary Care, University of Cambridge, Robinson Way, Cambridge, CB2 0SR UK; 4 Department of Primary Care and Public Health Sciences, King’s College London, London, SE1 3QD UK; 5 MRC Epidemiology Unit, Institute of Metabolic Science, Box 285, Addenbrooke’s Hospital, Hills Road, Cambridge, CB2 0QQ UK

**Keywords:** Behavior change techniques, Diet, Physical activity, Weight loss, Theory-based intervention, Intervention fidelity, Diabetes

## Abstract

**Background:**

Meta-analyses have identified promising behavior change techniques (BCTs) in changing obesity-related behaviors from intervention descriptions. However, it is unclear whether these BCTs are used by intervention participants and are related to outcomes.

**Purpose:**

The purpose of this study is to investigate BCT use by participants of an intervention targeting physical activity and diet and whether BCT use was related to behavior change and weight loss.

**Methods:**

Intervention participants (*N* = 239; 40–69 years) with recently diagnosed type 2 diabetes in the *ADDITION*-*Plus* trial received a theory-based intervention which taught them a range of BCTs. BCT usage was reported at 1 year.

**Results:**

Thirty-six percent of the participants reported using all 16 intervention BCTs. Use of a higher number of BCTs and specific BCTs (e.g., goal setting) were associated with a reduction in body mass index (BMI).

**Conclusions:**

BCT use was associated with weight loss. Future research should identify strategies to promote BCT use in daily life. (Trial Registration: ISRCTN99175498.)

**Electronic supplementary material:**

The online version of this article (doi:10.1007/s12160-014-9624-9) contains supplementary material, which is available to authorized users.

## Background

Health behavior change interventions typically teach participants a set of techniques (e.g., goal setting) to facilitate behavior change. To aid the design of new interventions and to optimize their efficacy, systematic reviews and meta-analyses have attempted to identify “active ingredients” of effective interventions. Several reviews have used taxonomies of behavior change techniques (BCTs) to classify intervention content and examine associations between BCTs and outcomes. For instance, the recently published Behavior Change Technique Taxonomy (v1) includes 93 behavior change techniques presented in 16 hierarchical clusters, which can be delivered by intervention providers or used by participants as part of self-management [1].

A systematic review of physical activity interventions for adults with type 2 diabetes [2] showed that interventions that included a higher number of BCTs were associated with better outcomes. However, a systematic review of dietary and physical activity interventions for obese adults with comorbidities [3] and a review of worksite physical activity interventions failed to find this association [4]. Reviews have also investigated whether specific BCTs are associated with larger effects. A meta-analysis using meta-regression found that self-monitoring in combination with at least one self-regulation technique, such as goal review, was related to diet and physical activity intervention effectiveness [5]. A meta-analysis of workplace physical activity interventions [6] identified specific goal setting and goal review to be associated with effectiveness among studies using fitness measures; although a similar, more recent review [4] found no significant relationship between individual BCTs and effectiveness. In a review of physical activity interventions targeting people with diabetes [2], no definitive list of effective BCTs could be identified, but the authors suggested some promising BCTs—these included prompting review of behavioral goals, planning social support and social change, goal setting, and barrier identification/problem solving. Interventions targeting physical activity (PA) and/or dietary behaviors, aimed at obese adults with obesity-related comorbidities or risk factors, were found to be more effective when including the provision of instructions, self-monitoring, relapse prevention, and prompting practice, as well as self-regulatory BCTs for dietary change [3]. A recent review investigating PA interventions among obese adults [7] identified more than 20 BCTs related to intervention effectiveness, including self-monitoring of behavioral outcome, social support/social change, teach to use prompts/cues, prompt practice, and prompt rewards contingent on effort or progress towards behavior. In sum, no clear picture of the most effective BCTs emerged, which may be due to differences across the reviews in included studies, covariates included in meta-regressions, types of outcomes, and BCT taxonomies used. On the whole, PA and diet change interventions including BCTs linked with self-regulation (e.g., goal setting, self-monitoring) have been found to be more effective than interventions which did not include these BCTs [8].

A major limitation with the reviews described above is that conclusions about the effectiveness of BCTs are based on intervention descriptions. However, the intervention that was delivered may differ from what was intended [9]. If intervention delivery is not assessed, or fidelity of delivery is low, it is difficult to interpret how the intervention achieved any effects and which components of the intervention may have contributed to its effectiveness. Furthermore, even with high fidelity of delivery, participants may respond differently to the intervention content than intended. For instance, participants may have been given a pedometer to monitor their walking but never used the pedometer or recorded the number of steps walked.

Participants’ use of BCTs in their daily life, also called “enactment” [10], is rarely measured. However, insight in participants’ BCT use could advance the evidence base about which techniques are effective in changing behavior and improving clinical outcomes. There is considerable variability in outcomes between participants of efficacious interventions, and it is important to establish which intervention components were most critical for behavior change.

There is some evidence about the associations between participants’ use of intervention strategies and changes in physical activity, dietary behaviors, and weight loss. For example, in a physical exercise intervention among adults, intervention engagement, measured by the time spent on an online intervention platform, predicted self-reported behavior change [11]. In a family-based behavioral weight control program, self-reported child and parent adherence to intervention components, such as recording food and calories, was related to better results [12]. In a weight loss program for overweight or obese adults, adherence to treatment components (e.g., attendance, self-monitoring) was associated with improvements in weight loss and biomarkers [13]. A recent study among participants who successfully lost weight found that “intentional strategies” for weight control, such as planning exercise, self-weighing, and writing down calorie content of food, were related to greater recent weight loss and changes in physical activity and dietary behaviors [14]. However, these studies only assessed a small number of BCTs or proxies for BCT use (e.g., time spent online), examined the effects of clusters of BCTs rather than individual BCTs [14], or assessed target behaviors (e.g., eating breakfast, decreasing fat intake, exercising) instead of BCT use (e.g. self-monitoring, planning) [12–14]. To our knowledge, no study has examined in the context of a trial of a behavior change intervention participants’ actual use of a wide range of BCTs for physical activity and dietary change which were taught in the intervention and whether BCT use is associated with self-reported and objectively measured behavior change and weight change over 1 year.

The current study focuses on the association between enactment of BCTs and behavioral and clinical outcomes among participants in the intervention arm of the *ADDITION*-*Plus* trial [15] who were recently diagnosed with type 2 diabetes. Type 2 diabetes is an increasing public health problem worldwide, and physical activity and healthy eating are important target behaviors in both the prevention and treatment of diabetes [16]. The *ADDITION*-*Plus* trial investigated whether adding a theory-based, individually-tailored behavior change intervention to intensive type 2 diabetes treatment improved health behavior change. The intervention did not change physical activity, dietary behaviors, and weight over and above intensive treatment alone, but participants in both study groups reported improvements in behavior (decreased fat intake, increased physical activity) over 1 year [17]. As not all intervention participants reported using all behavior change techniques taught in the intervention [17], heterogeneity in the enactment of BCTs might explain variance in behavioral and clinical outcomes among intervention participants.

The present study examines associations between the use of behavior change techniques and behavior change and weight loss.

## Aims

The aim of this study is to investigate whether reported use of behavior change techniques (BCTs) taught in a theory-based behavioral intervention is associated with change in objectively measured and self-reported physical activity (PA) and dietary behaviors and weight loss. More specifically:

1. Is the number of BCTs used by participants related to behavior change and weight loss? Does BCT use in the intervention group enhance weight loss and behavior change relative to the control group?
2. Which specific BCTs are associated with behavior change and weight loss?

## Methods

### Participants

Men and women (*N* = 478; 40–69 years) with a diagnosis of type 2 diabetes via routine clinical practice within the three previous years (*n* = 239) or via screening (*n* = 239) were recruited to the *ADDITION*-*Plus* trial (trial registration ISRCTN99175498) from 34 general practices in urban, suburban, and rural Cambridgeshire, East Hertfordshire, West Suffolk, and North Essex areas of England. The majority of the participants were white (97 % Caucasian ethnicity) and male (62 %), with a mean age of 60 years. Fifty-one percent of participants were in full- or part-time employment. All participants gave their informed consent prior to their inclusion in the study, and the procedures were in accordance with the Helsinki Declaration. Ethical approval was obtained from the Eastern Multi-Centre Research Ethics Committee (reference number 02/5/54).

Those randomized to the intervention (*n* = 239) received intensive diabetes treatment plus a facilitator-led, theory-based, individual-level behavior change intervention (*ADDITION*-*Plus*). The control group (*n* = 239) received intensive diabetes treatment only [15]. The results of the *ADDITION*-*Plus* trial have been published elsewhere [17]. In this study, the main focus is on evaluating BCT use among the intervention participants.

### Intervention Description

The *ADDITION*-*Plus* intensive diabetes treatment and behavior change intervention has been described in detail elsewhere [15]. All participants were offered intensive diabetes treatment over and above routine multidisciplinary primary care management of diabetes [15]. Participants were encouraged by their practice nurse and in written materials to achieve specific targets for health behavior change, e.g., increase physical activity gradually, decrease the consumption of fatty foods and sugar, and increase the consumption of fruit, vegetables, and whole grains. Participants with a body mass index (BMI) >28 kg/m^2^ were encouraged to lose 5–10 % of their body weight.

Intervention participants also received a behavior change intervention, based on psychological theory (theory of planned behavior, Carver and Scheier’s control theory, relapse prevention theory, and operant theory) and evidence [18–21]. The intervention was delivered by three trained lifestyle facilitators from outside the practice team; two had a professional background in nursing, and one in social work. Initial and refresher training took 7 days and covered the delivery of each session, including the theoretical basis and practising the BCTs. The facilitators used detailed protocols to guide each contact with the participant and received on-going supervision and feedback from a clinical psychologist. Facilitators were expected to teach participants a range of BCTs [15] to facilitate change and maintenance in physical activity and dietary intake and to promote medication adherence and smoking cessation, supported by a manual describing the BCTs. The operationalization of these BCTs is described in a supplementary table. Interested patients were offered pedometers. The intervention was delivered over 1 year at the participants’ general practice (or if necessary, participants’ homes or workplaces), including a 1-h introductory meeting followed by six 30-min meetings and four brief phone calls.

### Measures

Measurements were undertaken at outpatient clinical research facilities by trained staff following standard operational procedures and blind to participants’ study group allocation. Double data entry was undertaken by independent agencies, blind to the study group [15]. Measurement of weight, height, self-reported behaviors, and plasma vitamin C took place at baseline (before randomization) and at 1-year follow-up. Participants’ use of BCTs and physical activity were measured at 1 year.

#### Behavior Change Techniques

Participants in the intervention group completed a questionnaire, based on one that had been previously piloted and used in the ProActive trial [22], assessing their use of eight BCTs relating to increasing physical activity and eight BCTs relating to eating a lower-fat diet in the past 11 months (e.g., goal setting, action planning, self-monitoring; see Electronic Supplementary Material (ESM) Table 1). We used a binary scale (yes/no).

Principal component analysis (PCA), using the covariance matrix, was used to investigate whether BCTs could be grouped. After examining the scree plot, a solution with two components was best supported. Of the variance, 47.3 % was attributable to the use of BCTs in general (component 1), and 11.3 % was attributable to the contrast of using BCTs for either dietary change or PA change (component 2). In the rest of the paper, the focus is on the use of BCTs in general (component 1). As the PCA-rendered factor score correlated very highly with the composite score “number of BCTs used” (*r* = .998, *p* < .001), we decided to use the composite score for the analyses in this paper.

#### Physical Activity and Dietary Behaviors

Physical activity was assessed with a previously validated questionnaire (EPAQ2) [23] at baseline and 1 year and objectively at 1 year using a combined heart rate and movement sensor (Actiheart, CamNtech, Cambridge, UK) [24], which participants were asked to wear continuously for at least 4 days. The five participants with wear-time less than 48 h were excluded from analysis. The time-series data were summarized into physical activity energy expenditure (PAEE, in kJ/kg/day).

Consumption of a low-fat diet was measured as the fat percentage of total energy intake, derived from food frequency questionnaires (FFQ) [25] at baseline and 1 year. As an objective indicator of dietary quality, more specifically consumption of fresh fruit and vegetables, we measured plasma vitamin C at baseline and 1 year.

#### Adiposity

Height and weight were measured at baseline and 1 year, and body mass index (BMI) was calculated.

#### Demographic Variables

Participants’ age, socioeconomic status (SES, occupational class) [26], and sex were assessed by questionnaire at baseline.

### Statistical Analyses

We examined whether number of BCTs used and the proportion of dietary and PA BCTs differed across age groups, gender, and occupational status, using the Mann–Whitney *U* test and the median test in SPSS.

BCT use was heavily skewed, as a third of participants reported having used all 16 BCTs in the previous 11 months. In order to allow valid and interpretable analysis, BCT use was categorized into three groups of equal size: (1) those who reported using all 16 BCTs, (2) those who reported using 11–15 BCTs, and (3) those who reported using 0–10 BCTs. First, we used analysis of variance (ANOVA) to assess whether BMI in these groups differed at baseline.

Behavior-specific BCTs, i.e. *BCTs targeting physical activity* (*PA*) and *BCTs related to eating a lower*-*fat diet*, also showed skewness and a ceiling effect, so the following categorizations were made for each of them: (1) those who reported using all eight BCTs, (2) those who used six to seven BCTs, and (3) those who used zero to five BCTs. We first used ANOVA models to investigate the extent to which the PA- and diet-specific BCT user groups differed in self-reported physical activity and dietary behaviors, respectively, at baseline.

#### Question 1

We used analysis of covariance (ANCOVA) to compare change in BMI over 1 year (with calculated change scores) across the three BCT use groups (i.e., users of all 16 BCTs, users of 11–15 BCTs, and users of 0–10 BCTs), controlling for age, sex, baseline BMI,^1^ and SES. Next, we calculated associations between reported use of BCTs targeting physical activity (PA) and change in physical activity and between BCTs of eating a lower-fat diet and dietary change, respectively. ANCOVA models were used to investigate differences in PA change and dietary change, respectively, across these three groups of behavior-specific BCT use. Finally, we used ANCOVA to compare changes in BMI, physical activity, plasma vitamin C, and fat intake for the three BCT user groups with each other and also with the control group.

#### Question 2

We used ANCOVA to compare PA or dietary change between those who reported using and not using each individual BCT. We investigated whether BMI change differed across participants who used each individual BCT for (1) both PA and diet, (2) diet only, (3) PA only, or (4) neither. All ANCOVA analyses controlled for baseline BMI, gender, age, and SES. Eta-squared statistics are reported to indicate effect size. Analyses were conducted with SPSS (version 21).

## Results

### Descriptive Results

On average, BMI in the intervention group did not change significantly from baseline (mean (*M*) = 32.7, SD = 5.3) to 1 year (*M* = 32.1, SD = 5.2) [17]. Participants reported increases in physical activity and reductions in dietary fat intake, and plasma vitamin C levels increased over 1 year. Objectively measured physical activity energy expenditure was 35.2 kJ/kg/day (SD = 18.2) at 1 year.

Out of 239 intervention participants, 210 (87.9 %) participants provided valid data on all variables at 1 year. Of these, 75 participants (35.7 %) reported that they had used all 16 BCTs during the preceding 11 months. Table 1 shows the use of each BCT, which ranged from 61.5 % (BCTs “Preparing for/dealing with setbacks for PA” and “Using prompts/reminders for PA”) to 88.3 % (BCT “Goal setting for diet”). In total, 85 participants (40.5 %) reported that they had used all eight PA BCTs, and 97 participants (46.2 %) that they had used all eight dietary BCTs.

**Table 1:** Frequencies of behavior change technique (BCT) use and mean change in self-reported physical activity (PA) and fat intake and plasma vitamin C and objectively measured PA (controlling for baseline behavior, sex, age, socioeconomic status and baseline body mass index) in users and nonusers of specific BCTs

	Mean change (SD) in the outcome							
	BCT used for PA				BCT used for diet			
	No	Yes	Total	*p* value	No	Yes	Total	*p* value
Goal setting	34	187	221		26	196	222	
	15.40 %	84.60 %			11.70 %	88.30 %		
*n* in ANCOVA	33	182			26	189		
Self-reported (PA and fat %)	−0.31 (5.58)	1.10 (6.98)		0.070	0.50 (4.45)	−1.10 (6.24)		0.046
	*F*(1,210) = 3.47, *η* ^2^ = .016				*F*(1,208) = 4.03, *η* ^2^ = .019			
	30	175			25	177		
Objectively measured (PA and vit C)	32.43 (14.05)	34.84 (17.83)		0.533	2.63 (16.85)	1.37 (24.62)		0.676
	*F*(1,198) = .390, *η* ^2^ = .002				*F*(1,195) = .176, *η* ^2^ = .001			
Action planning	54	166	220		43	178	221	
	24.50 %	75.50 %			19.50 %	80.50 %		
	52	162			43	171		
Self-reported (PA and fat %)	−0.03 (6.99)	1.20 (6.71)		0.325	−0.12 (5.60)	−1.10 (6.19)		0.239
	*F*(1,207) = 0.972, *η* ^2^ = .005				*F*(1,207) = 1.39, *η* ^2^ = .007			
	50	155			38	163		
Objectively measured (PA and vit C)	35.75 (17.23)	34.23 (17.59)		0.919	−0.15 (15.88)	1.94 (25.35)		0.745
	*F*(1,198) = .010, *η* ^2^ = .000				*F*(1,194) = .106, *η* ^2^ = .001			
Using prompts/reminders	84	134	218		73	146	219	
	38.50 %	61.50 %			33.30 %	66.70 %		
	80	132			71	141		
Self-reported (PA and fat %)	0.74 (7.03)	0.96 (6.78)		0.407	−0.65 (6.37)	−1.09 (5.96)		0.643
	*F*(1,205) = .691, *η* ^2^ = .003				*F*(1,205) = .227, *η* ^2^ = .001			
	79	125			67	133		
Objectively measured (PA and vit C)	35.04 (18.03)	34.64 (17.17)		0.451	1.91 (22.20)	1.31 (24.77)		0.660
	*F*(1,197) = .571 *η* ^2^ = .003				*F*(1,193) = .194, *η* ^2^ = .001			
Motivating oneself	44	176	220		34	185	219	
	20 %	80 %			15.50 %	84.50 %		
	41	173			32	180		
Self-reported (PA and fat %)	−0.11 (5.01)	1.07		0.085	−0.56 (4.21)	−0.99 (6.34)		0.466
	*F*(1,207) = 2.99, *η* ^2^ = .014				*F*(1,205) = .533, *η* ^2^ = .003			
	40	165			30	169		
Objectively measured (PA and vit C)	32.85 (14.76)	35.21 (18.035)		0.152	0.80 (19.01)	1.71 (24.65)		0.715
	*F*(1,198) = 2.065, *η* ^2^ = .010				*F*(1,192) = .133, *η* ^2^ = .001			
Social support	81	139	220		58	162	220	
	36.80 %	63.20 %			26.40 %	73.60 %		
	79	135			57	156		
Self-reported (PA and fat %)	0.61 (6.26)	1.03 (7.15)		0.414	−0.07 (5.99)	−0.1.17 (6.10)		0.357
	*F*(1,207) = .669, *η* ^2^ = .003				*F*(1,206) = .852, *η* ^2^ = .004			
	75	130			55	145		
Objectively measured (PA and vit C)	33.64 (15.85)	35.04 (18.25)		0.099	0.84 (17.96)	1.52 (25.59)		0.487
	*F*(1,198) = 2.755, *η* ^2^ = .014				*F*(1,193) = .484, *η* ^2^ = .003			
Self-monitoring	64	156	220		70	151	221	
	29.10 %	70.90 %			31.70 %	68.30 %		
	61	153			68	146		
Self-reported (PA and fat %)	0.83 (4.83)	0.87 (7.49)		0.323	−.24 (6.02)	−1.20 (6.10)		0.082
	*F*(1,207) = .983, *η* ^2^ = .005				*F*(1,207) = 3.06, *η* ^2^ = .015			
					65	136		
Objectively measured (PA and vit C)	35.99 (16.22)	34.06 (17.98)		0.141	−0.44 (18.48)	2.49 (26.01)		0.181
	*F*(1,198) = .141, *η* ^2^ = .001				*F*(1,194) = 1.799, *η* ^2^ = .009			
Goal review	69	152	221		67	154	221	
	31.20 %	68.80 %			30.30 %	69.70 %		
	67	148			65			
Self-reported (PA and fat %)	1.05 (5.52)	0.74 (7.35)		0.820	1.02 (5.57)	−1.74 (6.12)		0.001
	*F*(1,208) = .052, *η* ^2^ = .000				*F*(1,207) = 11.44, *η* ^2^ = .052			
	65	141			64	137		
Objectively measured (PA and vit C)	35.86 (17.01)	34.11 (17.67)		0.620	−2.92 (22.89)	3.63 (24.05)		0.042
	*F*(1,198) = .247, *η* ^2^ = .001				*F*(1,194) = 4.19, *η* ^2^ = .021			
Preparing for/dealing with setbacks	85	136	221		73	148	221	
	38.50 %	61.50 %			33 %	67 %		
	81	134			71	143		
Self-reported (PA and fat %)	0.73 (5.84)	0.91 (7.37)		0.542	0.11 (5.96)	−1.40 (6.09)		0.021
	*F*(1,208) = .374, *η* ^2^ = .002				*F*(1,207) = 5.39, *η* ^2^ = .025			
	79	127			68	133		
Objectively measured (PA and vit C)	37.04 (17.97)	33.18 (17.01)		0.385	−0.71 (21.46)	2.70 (24.95)		0.279
	*F*(1,199) = .757, *η* ^2^ = .004				*F*(1,194) = 1.18, *η* ^2^ = .006			

Self-reported PA is reported as total METs per day

The number of BCTs used was not statistically significantly different between age groups (*p* = .061), occupational status (*p* = .068), and gender (*p* = .069). However, individuals who used 0–10 BCTs had a lower BMI at baseline than those using 11–15 BCTs (*M*(SD) = 31.2 kg/m^2^ (4.29) vs. *M*(SD) = 33.6 kg/m^2^ (5.14) (*F*(2,206) = 4.233, *p* = .016). Participants in the three PA BCT groups did not differ in baseline BMI (*F*(2,209) = 1.992, *p* = .139), but those in the dietary BCT groups did (*F*(2,212) = 4.496, *p* = .012): participants using all eight dietary BCTs had higher BMI (*M* = 33.81 kg/m^2^ (SD = 5.98)) than those using zero to five BCTs (*M* = 31.26 kg/m^2^ (SD = 4.40)). PA BCT groups did not differ in baseline self-reported activity levels (*F*(2,209) = .825, *p* = .440), and dietary BCT groups did not differ in baseline plasma vitamin C levels (*F*(2,202) = 1.376, *p* = .255) or self-reported fat intake (*F*(2,211) = .087, *p* = .916) at baseline.

### Is the Number of BCTs Used Related to Weight Loss and Behavior Change?

**Fig. 1:**
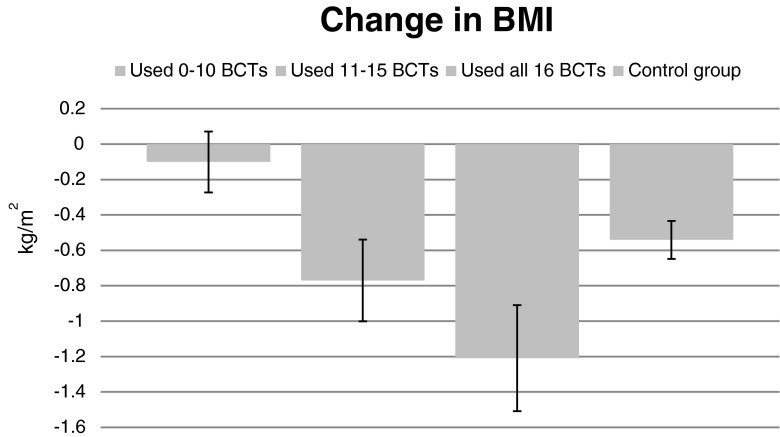
Mean change in body mass index (BMI) in intervention participants (*N* = 204) according to behavior change technique (BCT) use, (0–10 BCTs, *n* = 69; 11–15 BCTs, *n* = 64; all 16 BCTs, *n* = 71) and control group (*N* = 218)

The number of BCTs used was significantly associated with a decrease in BMI over 1 year (*F*(2,197) = 3.139, *p* = .045, *η* ^2^ = .031, adjusted for baseline BMI). Individuals who reported using all BCTs lost more weight (*M* = −1.18 kg/m^2^ (SD = 2.55)) compared to those who used 10 or fewer BCTs (*M* = −.10 kg/m^2^ (SD = 1.42), *p* = .013). BMI reduction among those using all BCTs was significantly greater (mean difference = 0.59 kg/m^2^, SE = .24, *p* = .014) than among the *ADDITION*-*Plus* control group participants (*F*(4,426 = 2.969, *p* = .019, *η* ^2^ = .027, adjusted for baseline BMI) (Fig. 1).

Comparing change in self-reported physical activity between the BCT user groups (in the intervention group) and control group, ANCOVA indicated an association with BCT use (*F*(4,431 = 2.884, *p* = .022, *η* ^2^ = .026): those using 11–15 BCTs differed significantly from those using fewer BCTs and from the control group, with increases of on average 2.4 (SD = 6.63) METs per day.

The BCT groups did not differ from the control group with regard to changes in plasma vitamin C (*F*(4,399 = 1.250, *p* = .289, *η* ^2^ = .012) or fat intake (*F*(4,428 = 1.433, *p* = .222, *η* ^2^ = .013).

The same patterns emerged when the behavior-specific BCT groups were investigated, with the users of six to seven PA BCTs increasing their PA significantly more than the control group (*F*(4,431 = 2.653, *p* = .033, *η* ^2^ = .024). There was no difference between users of diet-specific BCTs and the control group.

The number of PA BCTs used was significantly related to BMI reduction among the intervention participants (*F*(2,200 = 4.730, *p* = .010, *η* ^2^ = .045): those who used all eight PA BCTs lost more weight (*M* = −1.01 kg/m^2^, SD = 2.45) than those who used zero to five PA BCTs (*M* = .04, SD = 1.34). The number of diet BCTs was unrelated to BMI change *F*(2,203 = 1.128, *p* = .326, *η* ^2^ = .011).

Within the intervention group, the number of PA BCTs used was unrelated to change in self-reported PA over the previous year (*F*(2,201) = 1.188, *p* = .307, *η* ^2^ = .012) or 1-year objective physical activity levels (*F*(2,193) = .081, *p* = .922, *η* ^2^ = .001) (controlling for baseline self-reported PA for both set of analyses). Similarly, the number of diet BCTs was unrelated to self-reported change in fat intake (*F*(2,203) = 1.445, *p* = .238, *η* ^2^ = .014) or plasma vitamin C over the previous year (*F*(2,189) = .224, *p* = .800, *η* ^2^ = .002).

### Which BCTs are Associated with Weight Loss and Behavior Change?

We compared BMI change between those who used each individual BCT for (1) both PA and diet, (2) diet only, (3) PA only, or (4) neither, controlling for baseline BMI and other variables. The main effects were significant in participants who used goal setting, goal review, and social support (see Table 2).^2^ Specifically, those who used goal setting both to eat a lower-fat diet and increase physical activity lost significantly more weight (*M* change in BMI = −.88 kg/m^2^, SD = 2.13) than those who used goal setting for dietary change only (*M* = .08 kg/m^2^, *SD* = 1.50, *p* = .029, *N* = 21) or for neither behavior (*M* = .49 kg/m^2^, SD = .98, *p* = .023, *N* = 13). Participants who had used social support to change both behaviors lost more weight than those who used it for dietary change only or neither behavior. Those who used goal review for both behaviors lost more weight than those who had used goal review to increase physical activity only or for neither behavior (see Table 3).

**Table 2:** Analysis of covariance: Differences in body mass index change between the four behavior change technique (BCT) user groups (BCT used for physical activity and diet, diet only, physical activity only, neither) for each BCT

		*p*	*η* ^2^
Goal setting	*F*(3,206) = 3.476	0.017	0.048
Action planning	*F*(3,205) = 1.749	0.158	0.025
Using prompts/reminders	*F*(3,204) = .6230	0.601	0.009
Motivating oneself	*F*(3,203) = 2.250	0.084	0.032
Social support	*F*(3,204) = 3.867	0.010	0.054
Self-monitoring	*F*(3,205) = 2.028	0.111	0.029
Reviewing goals	*F*(3,206) = 2.751	0.044	0.039
Preparing for/dealing with setbacks	*F*(3,206) = 1.870	0.136	0.027

**Table 3:** Body mass index (BMI) change in the four behavior change technique (BCT) user groups (mean (SD), with the group that used the BCT for both behaviors as the reference group) (analysis of covariance)

	Nonusers	Used for PA only	Used for diet only	Used for both (ref)
Goal setting	*n* = 13	*n* = 13	*n* = 21	*n* = 167
*M* BMI change (SD)	0.49 (0.97)*	0.23 (1.24)	0.08 (1.50)*	−0.88 (2.13)
Action planning	*n* = 28	*n* = 14	*n* = 25	*n* = 146
*M* BMI change (SD)	0.17 (1.43)*	−0.08 (1.18)	−0.63 (1.61)	−0.87 (2.20)
Prompt use	*n* = 61	*n* = 10	*n* = 21	*n* = 120
*M* BMI change (SD)	−0.61 (1.65)	−0.42 (1.19)	−0.24 (1.59)	−0.79 (2.29)
Motivating	*n* = 22	*n* = 9	*n* = 18	*n* = 162
*M* BMI change (SD)	0.38 (1.40)*	−0.66 (1.40)	−0.19 (1.26)	−0.85 (2.16)
Social support	*n* = 46	*n* = 10	*n* = 32	*n* = 124
*M* BMI change (SD)	−0.01 (1.66)**	−0.63 (2.11)	−0.03 (1.45)*	−1.03 (2.20)
Self-monitoring	*n* = 46	*n* = 22	*n* = 15	*n* = 130
*M* BMI change (SD)	0.02 (1.53)*	−0.65 (2.25)	−0.26 (0.90)	−0.92 (2.19)
Goal review	*n* = 51	*n* = 14	*n* = 15	*n* = 134
*M* BMI change (SD)	−0.06 (1.43)*	0.47 (1.75)*	−0.67 (1.33)	−0.98 (2.22)
Preparing for/dealing with setbacks	*n* = 56	*n* = 14	*n* = 26	*n* = 118
*M* BMI change (SD)	0.06 (1.58)*	−0.84 (1.91)	−0.62 (1.27)	−0.96 (2.28)

*p < .05; **p < .01 (significant differences in BMI change compared to the reference group (those who used the BCT for both behaviors))

Post hoc group comparisons indicated that participants who used action planning, motivating oneself, self-monitoring, and preparing for setbacks for both behaviors lost significantly more weight than those who used these BCTs for neither behavior.

The results for ANCOVAs are displayed in Table 1. Participants’ use of specific physical activity BCTs was unrelated to change in self-reported physical activity and levels of objectively measured physical activity at 1 year. Participants who set goals reported larger increases in self-reported PA than those who did not, albeit not achieving statistical significance (*p* = .07).

**Fig. 2:**
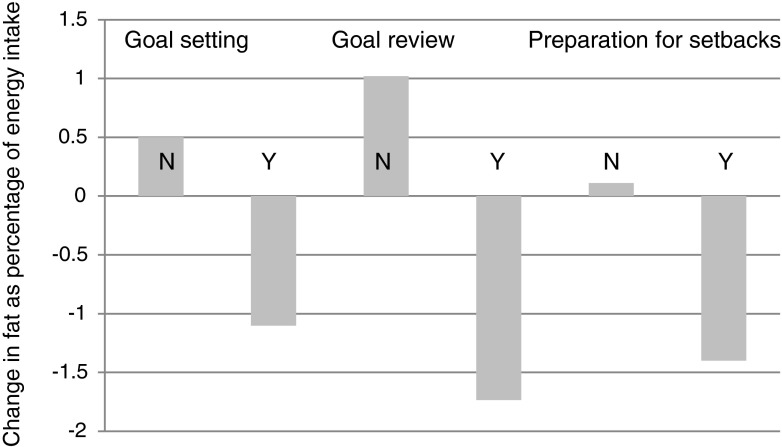
Mean changes in self-reported fat intake (% of calorie intake) over 1 year among users (*Y* = Yes) and nonusers (*N* = No) of goal setting, goal review, and preparing for/dealing with setbacks (all baseline-adjusted differences significant *p* < .05)

Use of goal setting, goal review, and preparing for/dealing with setbacks in order to eat a lower-fat diet was significantly (*p* < .05) associated with decreases in self-reported fat intake over the previous year (see Fig. 2). Self-monitoring almost reached significance (*p* = .082). Use of goal review to eat a lower-fat diet was significantly related to increases in plasma vitamin C (nonusers *M* change = −2.92, SD = 22.89, users *M* = 3.63, SD = 24.05, *p* = .042).

## Discussion

Only one third of *ADDITION*-*Plus* intervention participants with recently diagnosed type 2 diabetes used all 16 BCTs taught in the intervention. Those who reported having used all BCTs showed larger reductions in BMI than those who used 10 or fewer BCTs. Specifically, a higher number of BCTs used for physical activity change was related to larger reductions in BMI. Interestingly, the number of BCTs used was unrelated to change in PA or dietary behaviors. Of the individual BCTs, use of goal setting, goal review, and social support for both behaviors showed the strongest relationship with BMI reduction. No single BCT was significantly related to PA change. Participants who used goal setting, goal review, and prepared for and dealt with setbacks in relation to eating a lower fat diet reported larger reductions in self-reported fat intake. In summary, techniques consistent with control theory [20] as well as social support emerged as promising BCTs in weight control.

Overall, the *ADDITION*-*Plus* trial had procedures to ensure high fidelity, e.g., facilitators used detailed protocols for each contact and received on-going supervision and feedback. However, the present study showed that in terms of “enactment,” only one third of the participants reported using all the BCTs taught in the intervention. Had more participants enacted all the BCTs for both behaviors, as intended, the intervention may have achieved a greater effect.

Our findings are partially in line with evidence from systematic reviews and meta-analyses of PA and dietary behavior change interventions among adults, which identified techniques related to goal management [2, 3, 5, 6, 8] and social support [2, 3, 7, 8] as promising. Contrary to primary studies, e.g., [12–14] and reviews, e.g., [5], which identified self-monitoring as effective, in our study, it was unrelated to behavior change and weight loss. This may be due to inadequate power in part arising from our dichotomous measure of BCT use (yes/no); a measure of frequency of use might have shown stronger associations with behavior change.

No BCTs were associated with self-reported and objectively measured physical activity, although use of PA BCTs was related to weight loss. Our study also failed to find associations between use of prompts/cues and PA change contrary to a review of PA interventions among obese individuals [7]. We measured PA change over 1 year, while many studies included in the above systematic reviews have shorter follow-ups. Furthermore, self-reported physical activity is subject to recall, social desirability, and weight-dependent bias. Participants might not be able or willing to accurately report their activity, especially as the EPAQ2 used in our study measures physical activity during the preceding 12 months. As the self-reported PA measure we used includes the time when participants only made initial changes in their physical activity, it might not be the most appropriate measure to assess physical activity at 1 year post-intervention. Furthermore, although objective measurement of PA is more reliable and less prone to bias than self-report, the objective measure only reflects PA behavior over 4 days at 1 year, which may not be representative of usual PA levels. This may be partly due to demand effects, and the absence of a baseline measure, which precluded adjustment for potential confounding arising from differences in the BCT groups’ objectively measured activity at baseline. Also, we measured energy expenditure rather than the specific behaviors targeted in the intervention, e.g., walking or cycling. However, as typical PA and dietary behavior during the past year was the target of interest, the most objective, reliable, and valid indicator of any changes in the participant’s energy balance behaviors during the past year would be change in BMI. Perhaps the reason that the association was significant for BMI is that it is measured with greater precision than all the other outcomes.

This exploratory process evaluation study has several strengths. To our knowledge, this is the first study to investigate participants’ use of a wide range of specific BCTs and associations with behavior change and weight loss. The study assessed a variety of outcome measures, including self-report and objective measures of energy-balance behaviors (physical activity and diet), and BMI.

The study also has its limitations. First, due to the small sample size, the risk of type II errors exists. For this reason, it was also not possible to control for the effects of using other BCTs or combinations of BCTs. On the other hand, some associations may be due to chance because of the large number of significance tests conducted. Also problematic is the low sample size for some subgroup analyses, especially for PA, which tends to have very high variance. Secondly, BCT use was measured with self-reported dichotomous items. The frequency or extent to which particular BCTs are used (e.g., number of days per week) may reveal more variability in responses and mediate intervention effectiveness, e.g., [27]. Furthermore, some BCT labels (e.g., “using prompts and reminders”) may have been unclear. Third, BCT use was measured retrospectively at 1-year follow-up, so the causal direction of the effects is unclear: Perhaps participants who lost more weight were more likely to have reported using more BCTs, and those who did not lose weight reported not having used BCTs although they had tried. Future studies could use measurements during the intervention, using approaches such as ecological momentary assessment (despite its disadvantages such as measurement burden and required resources), to increase reliability and validity. Furthermore, even if measurement of BCT use were reliable, perhaps participants who used most BCTs and certain specific BCTs also engaged in another behavior or possessed an individual characteristic that was not measured but which helped to lose weight *and* was correlated with the BCT use (e.g., attendance at all sessions).

Fourth, the comparison with the *ADDITION*-*Plus* control group should be interpreted with caution as we did not assess BCT use in this group. It is likely that participants in the control group used some BCTs spontaneously. They were encouraged to increase physical activity, eat a healthier diet, and lose weight during contacts with the practice team and may have set goals and made action plans to change these behaviors as a result. Fifth, it is worth noting that levels of BCT enactment by patients might be lower in routine primary care if the intervention were offered by practitioners with limited or no training in facilitating behavior change.

Which BCTs seem to work best? These findings indicate that for weight loss, using more BCTs and specifically physical activity-related BCTs may be beneficial. Advising on, arranging, or providing social support for both PA and dietary change can also be recommended for weight loss. Setting and reviewing goals appeared useful for weight loss and dietary fat intake reduction, and preparing for and dealing with setbacks for reducing dietary fat intake. The findings have implications for future research and clinical practice. We recommend more research to identify the modifiable determinants of BCT use in daily life (e.g., engagement, attitudes, intention) and to evaluate strategies to promote BCT enactment. Potential strategies include, for example, asking participants to commit themselves to set and review goals regularly, demonstrating BCTs during intervention contacts, informing participants about the evidence linking BCT use to weight loss, and providing feedback on BCT use in subsequent contacts. We recommend that future intervention studies assess participant enactment of intervention BCTs as part of process evaluation, using reliable measures of frequency of BCT use and assessing participants’ perceptions of usefulness of individual BCTs and their optimal frequency of use. This has the potential to increase our understanding of mechanisms of effect and improve the efficiency of future interventions.

In terms of clinical practice, as use of a higher number of BCTs was related to weight loss, future interventions should provide a toolbox of BCTs rather than teach a few BCTs only. Also, practitioners could encourage participants to use as many BCTs as possible on the basis of evidence that people who use recommended BCTs are more likely to achieve weight loss.

This is one of the first studies to investigate use of BCTs among people with recently diagnosed type 2 diabetes who took part in a theory-based behavior change intervention. We demonstrated that use of a higher number of BCTs was significantly related to weight loss. Use of goal setting, goal review, and preparing for and dealing with setbacks was related to significant reductions in reported dietary fat intake. The use of goal setting, goal review, and social support to change both PA and dietary fat intake was related to reductions in BMI. Our study demonstrates that measures of enactment have the potential to add value to the process evaluation of behavior change intervention studies.

## Electronic supplementary material

Below is the link to the electronic supplementary material.

ESM 1 (PDF 87 kb)

## Footnotes

1 The three BCT groups were formed on completion of the intervention and after having observed the distribution of the data, with the aim of creating three equally sized groups. As differences in outcome may have been attributable to differences in baseline between these three groups, ANCOVA was used to correct for baseline difference, allowing BCT associations with outcomes to be controlled for any baseline group differences. Baseline differences in BCT groups were observed to range from being of negligible size to being statistically significant for BMI.

2 The BCT-user groups differed in baseline BMI significantly for four BCTs (using prompts/reminders, self-monitoring, goal review, and preparing for/dealing with setbacks). When the same ANCOVAs were calculated without controlling for baseline BMI, motivating oneself, self-monitoring, and preparing for/dealing with setbacks were significantly associated with BMI change.

## References

[1] Michie S, Richardson M, Johnston M, et al. The behavior change technique taxonomy (v1) of 93 hierarchically clustered techniques: Building an international consensus for the reporting of behavior change interventions. *Ann Behav Med.* 2013:1*–*15. 10.1007/s12160-013-9486-6 23512568

[2] Avery L, Flynn D, van Wersch A, Sniehotta FF, Trenell MI (2012). Changing physical activity behavior in type 2 diabetes: A systematic review and meta-analysis of behavioral interventions. Diabetes Care.

[3] Dombrowski SU, Sniehotta FF, Avenell A (2012). Identifying active ingredients in complex behavioural interventions for obese adults with obesity-related co-morbidities or additional risk factors for co-morbidities: A systematic review. Health Psychol Rev.

[4] Taylor N, Conner M, Lawton R (2011). The impact of theory on the effectiveness of worksite physical activity interventions: A meta-analysis and meta-regression. Health Psychol Rev.

[5] Michie S, Abraham C, Whittington C, McAteer J, Gupta S (2009). Effective techniques in healthy eating and physical activity interventions: A meta-regression. Health Psychol.

[6] Abraham C, Graham-Rowe E (2009). Are worksite interventions effective in increasing physical activity? A systematic review and meta-analysis. Health Psychol Rev.

[7] Olander E, Fletcher H, Williams S (2013). What are the most effective techniques in changing obese individuals’ physical activity self-efficacy and behaviour: A systematic review and meta-analysis. Int J Behav Nutr Phys Act.

[8] Greaves C, Sheppard K, Abraham C (2011). Systematic review of reviews of intervention components associated with increased effectiveness in dietary and physical activity interventions. BMC Public Health.

[9] Hardeman W, Michie S, Fanshawe T (2007). Fidelity of delivery of a physical activity intervention: Predictors and consequences. Psychol Health.

[10] Bellg AJ, Borrelli B, Resnick B (2004). Enhancing treatment fidelity in health behavior change studies: Best practices and recommendations from the NIH Behavior Change Consortium. Health Psychol.

[11] Richert J, Lippke S, Ziegelmann JP (2011). Intervention–engagement and its role in the effectiveness of stage-matched interventions promoting physical exercise. Res Sports Med.

[12] Wrotniak BH, Epstein LH, Paluch RA, Roemmich JN (2005). The relationship between parent and child self-reported adherence and weight loss. Obes Res.

[13] Acharya SD, Elci OU, Sereika SM (2009). Adherence to a behavioral weight loss treatment program enhances weight loss and improvements in biomarkers. Patient Prefer Adherence.

[14] Fuglestad P, Jeffery R, Sherwood N (2012). Lifestyle patterns associated with diet, physical activity, body mass index and amount of recent weight loss in a sample of successful weight losers. Int J Behav Nutr Phys Act.

[15] Griffin S, Simmons R, Williams K (2011). Protocol for the ADDITION-Plus study: A randomised controlled trial of an individually-tailored behaviour change intervention among people with recently diagnosed type 2 diabetes under intensive UK general practice care. BMC Public Health.

[16] Paulweber B, Valensi P, Lindström J (2010). A European evidence-based guideline for the prevention of type 2 diabetes. Horm Metab Res.

[17] Griffin SJ, Simmons RK, Prevost AT (2014). Multiple behaviour change intervention and outcomes in recently diagnosed type 2 diabetes: The ADDITION-Plus randomised controlled trial. Diabetologia.

[18] Marlatt GA, Gordon JR (1985). Relapse Prevention: Maintenance Strategies in the Treatment of Addictive Behaviors.

[19] Ajzen I (1991). The theory of planned behavior. Organ Behav Hum Decis Process.

[20] Carver CS, Scheier MF, Wyers RS (1999). Themes and issues in the self-regulation of behavior. Perspectives on Behavioral Self-Regulation. Advances in Social Cognition.

[21] Kazdin AE (2001). Behavior Modification in Applied Settings.

[22] Kinmonth A-L, Wareham NJ, Hardeman W (2008). Efficacy of a theory-based behavioural intervention to increase physical activity in an at-risk group in primary care (ProActive UK): A randomised trial. Lancet.

[23] Wareham N, Rennie K (1998). The assessment of physical activity in individuals and populations: Why try to be more precise about how physical activity is assessed?. Int J Obes Relat Metab Disord.

[24] Brage S, Brage N, Franks P, Ekelund U, Wareham N (2005). Reliability and validity of the combined heart rate and movement sensor Actiheart. Eur J Clin Nutr.

[25] Bingham S, Gill C, Welch A (1997). Validation of dietary assessment methods in the UK arm of EPIC using weighed records, and 24-hour urinary nitrogen and potassium and serum vitamin C and carotenoids as biomarkers. Int J Epidemiol.

[26] Office for National Statistics: *The National Statistics Socio-economic Classification (NS-SEC rebased on the SOC2010).* Retrieved 23.6., 2013 from http://www.ons.gov.uk/ons/guide-method/classifications/current-standard-classifications/soc2010/soc2010-volume-3-ns-sec--rebased-on-soc2010--user-manual/index.html.

[27] VanWormer JJ, Linde JA, Harnack LJ, Stovitz SD, Jeffery RW (2012). Self-weighing frequency is associated with weight gain prevention over 2 years among working adults. Int J Behav Med.

