# Supplementary material for: Which Behavior Change Techniques are Associated with Changes in Physical Activity, Diet and Body Mass Index in People with Recently Diagnosed Diabetes?
Source: Ann Behav Med. 2014 May 8;49(1):7–17. doi: 10.1007/s12160-014-9624-9 (PMC4335098; doi:10.1007/s12160-014-9624-9)
Supplement: Supplementary file 1 — (PDF 87 kb) [file 12160_2014_9624_MOESM1_ESM.pdf]

Electronic Supplementary Material Table 1.

The operationalization of behavior change techniques in the ADDITION-Plus intervention

| **Behaviour change technique** | **Operationalization in the intervention** |
|---|---|
| Goal setting | Prompt participants to set a behavioral goal (e.g., to increase walking, to eat more fruit). |
| Action planning | Prompt participants to define where and when they will act on their goal, and what preparations and support are needed. |
| Using prompts/reminders | Prompt participants to identify a reminder that will prompt them to act on their goal (e.g., stick their action plan on the fridge door). |
| Motivating oneself | Prompt participants to identify positive things they will get out of achieving goals, and to anticipate problems and work around them. Encourage participants to reward themselves when they implement their action plan, e.g., buy a special magazine, elicit praise from family, and highlight positive feelings. Encourage participants to remain motivated after setbacks and get back on track as soon as possible. Encourage participants to engage in positive self-talk. Prompt participants to elicit support from others. |
| Social support | Prompt participants to identify people who could support them in achieving their goal, e.g., friends, family and health |

| | |
|---|---|
| | practitioners. Introduce ideas on how to elicit support. Discuss how participants could deal with people who are not supportive. |
| Self-monitoring | Ask participants what they want to monitor, and encourage them to use weekly self-monitoring forms. |
| Reviewing goals | Prompt participants to review progress with their goal and action plan, informed by self-monitoring forms if completed. Ask about any benefits and downsides, support received, and facilitators and barriers. Prompt participants to revise their goal(s). |
| Preparing for and dealing with setbacks | Ask participants to identify any situations which might make it difficult to put their plan into action, and identify strategies to deal with these potential situations. Encourage participants to react in a helpful way to setbacks when they occur (i.e. not feel guilty, see them as a normal part of changing behaviour) and use them as an opportunity to review goals and make a new action plan. Encourage participants to consider how to get back on track after a setback. |
